# The prognostic significance of *MCL1* copy number gain in esophageal squamous cell carcinoma

**DOI:** 10.18632/oncotarget.21181

**Published:** 2017-09-23

**Authors:** Chen Xu, Yalan Liu, Jie Huang, Hao Wang, Lijie Tan, Yifan Xu, Zhengzeng Jiang, Xin Wang, Yingyong Hou, Dongxian Jiang, Qun Wang

**Affiliations:** ^1^ Department of Pathology, Zhongshan Hospital, Fudan University, Shanghai 200032, P. R. China; ^2^ Department of Thoracic Surgery, Zhongshan Hospital, Fudan University, Shanghai 200032, P. R. China; ^3^ Department of Pathology, School of Basic Medical Sciences & Zhongshan Hospital, Fudan University, Shanghai 200032, P. R. China

**Keywords:** MCL1 copy number gain, prognostic marker, lymph node metastasis, clinical stage, ESCC

## Abstract

**Background:**

*MCL1* copy number variations have been reported to be associated with cancer prognosis in several cancers. However, the role of *MCL1* gain has not yet been determined in esophageal squamous cell carcinomas (ESCC).

**Methods:**

Fluorescence *in situ* hybridization (FISH) for *MCL1* was performed on 262 ESCC samples using tissue microarray (TMA).

**Results:**

The median age of ESCC patients was 62 years (range 37–83), with frequencies between women (16.4%) and men (83.6%). Of the 262 tumors, 77 tumors (29.4%) had high *MCL1* gain. In the multivariate analysis, lymph node metastasis (HR: 3.236, *P*<0.001 for DFS; HR: 3.501, *P*<0.001 for OS) and clinical stage (HR: 3.388, *P*<0.001 for DFS; HR: 3.616, *P*<0.001 for OS) were identified as independent worse prognostic factors. Interestingly, among patients without lymph node metastasis or stage I-II patients, high *MCL1* gain was associated with better DFS (*P*=0.009 or 0.046) and OS (*P*=0.014 or 0.069) after disease free survival time was more than or equal to 12 months. Reversely, among patients with lymph node metastasis or stage III-IVa patients, high *MCL1* gain was associated with poorer DFS (*P*=0.007 or 0.021) and OS (*P*=0.029 or 0.068) after disease free survival time was more than or equal to 29 months.

**Conclusion:**

We observed that high *MCL1* gain had bidirectional prognostic significance in ESCC patients with different lymph node status or clinical stage. These findings might provide the useful way of detailed risk stratification in patients with ESCC, and an insight into pathogenesis and mechanism of progression in ESCC.

## INTRODUCTION

Esophageal carcinoma is the sixth leading cause of cancer-related mortality and the eighth most common cancer worldwide [1]. In China, the incidence is approximately 478, 000, and the mortality is 375,000 in 2015, being the third most commonly diagnosed cancers and fourth leading causes of cancer death [2]. And more than 95% of all esophageal cancers in China are esophageal squamous cell carcinomas (ESCC) [3]. Although the advance of surgery, radiotherapy, and chemotherapy has improved the survival of ESCC patients in recent years, the long-term survival rate still needs to be improved. Although TNM classification lays the foundation for ESCC prognostic management, it does not provide sufficient information about biological tumor progression [1]. There is demand for revealing molecular markers that could predict patients’ survival.

To limit or circumvent apoptosis is recognized as one of the fundamental features of cancer. B-cell lymphoma 2 (Bcl-2) family proteins have preeminent importance in the mitochondrial apoptotic pathway and are characterized by the presence of anti-apoptotic and pro-apoptotic proteins [4]. Myeloid cell leukemia sequence 1 (*MCL1*), located in 1q21.2, is a Bcl2 anti-apoptotic member, which could block apoptosis induced by various apoptosis-inducing stressors, such as DNA damage, hypoxia or oncogenic signaling [5]. Studies using targeted gene deletion, RNA interference or inducible expression have shown that Mcl1 is essential for the growth of diverse tumors, including acute myeloid leukaemia [6], lymphomas [7], papillary thyroid carcinoma [8], breast cancers [9], oral squamous cell carcinomas [10], and non-small-cell lung carcinoma [11]. These results provided information to substantiate the possibility of Mcl1 as a clinically useful indicator in the prognosis of cancer.

Mcl1 alteration occurs through various mechanisms, including chromosomal translocation, gene amplification, and signal transduction alterations associated with transformation [12]. Gene copy number gain or amplification of *MCL1* is frequently found in solid tumors [13]. What is more, *MCL1* copy number variations have been reported to be associated with cancer prognosis in papillary thyroid carcinoma [8] and non-small-cell lung carcinoma [11]. Nevertheless, there have been rare studies addressing the roles of *MCL1* copy number variations in ESCC outcomes.

In this study, we detected *MCL1* copy number variation in 262 ESCC using tissue microarrays, and searched for correlations between *MCL1* copy number gain and prognosis in ESCC; additionally, we compared it in patients with different lymph node status and clinical stage.

## RESULTS

### Clinicopathologic characteristics of ESCC patients

The clinicopathologic features of a total of 262 cases of ESCC were summarized in Table 1. Briefly, median age was 62 years (range 37–83), with frequencies between women (16.4%, 43 out of 262) and men (83.6%, 219 out of 262). Former or current smokers represented 40.8% of all patients. The location of the tumor in 49.4% of patients was middle esophagus, in lower was 45.5% and in upper was only 5.1%. On the basis of the AJCC Staging Manual (seventh edition), 58.4% cases were histologically graded as well to moderately differentiated, and 41.6% were poorly differentiated. Vessel and nerve involvement were identified in 47 (17.9%) and 70 (26.7%) tumors, respectively. Lymph node metastasis was identified in 127 (48.5%) patients. Stage I-II cases accounted for 59.5% (156 out of 262), while stage III-IVa cases were 40.5% (106 out of 262), respectively.

**Table 1:** Correlation between high *MCL1* gain and clinico-pathological features in full cohort of ESCC

	Number	High *MCL1* gain	
		Yes	*P* value
Sex			0.894
Female	43	13	
Male	219	64	
Age			0.404
<60	109	29	
≥60	153	48	
Grade			0.415
I+II	153	42	
III	109	35	
Invasive depth			0.518
I	13	2	
II	68	20	
III	181	55	
Vessel involvement			0.260
No	215	60	
Yes	47	17	
Nerve involvement			0.457
No	192	54	
Yes	70	23	
Lymph node metastasis			0.930
No	135	40	
Yes	127	37	
Site			0.314
up	13	3	
middle	125	33	
down	115	40	
Smoking			0.500
No	155	48	
Yes	107	29	
Clinical stage			0.610
I-II	156	44	
III-IVa	106	33	
Disease progression			0.729
No	108	33	
Yes	154	44	
Death of esophageal cancer			0.445
No	113	36	
Yes	149	41	

Invasive Depth, I confined to submucosal layer; II invasion of muscular layer, III beyond the muscularis.

### *MCL1* copy number variation in ESCC

**Figure 1:**
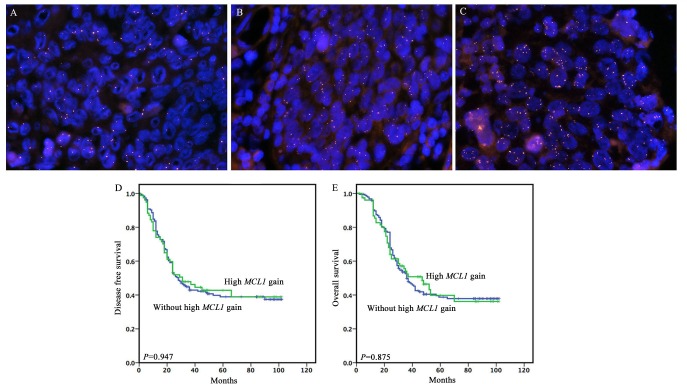
Representative fluorescence *in situ* hybridization (FISH) signal patterns of select *MCL1* anomalies and the prognostic significance of high *MCL1* gain in full cohort **(A)** Normal or loss of *MCL1*, **(B)** low *MCL1* gain (2.5 to 5 average *MCL1* copies/nucleus), **(C)** High *MCL1* gain (>5.0 average *MCL1* gene copies/nucleus), (**D** and **E**) High *MCL1* gain for DFS and OS.

Of the 262 tumors, 77 tumors (29.4%), 95 tumors (36.3%) and 90 tumors (34.3%) had >5.0, 2.5 to 5 and <2.5 average *MCL1* copies/nucleus, respectively. Figure 1 illustrates representative FISH signal patterns of select *MCL1* anomalies including high *MCL1* gain (>5.0 average *MCL1* gene copies/nucleus), low *MCL1* gain (2.5 to 5 average *MCL1* copies/nucleus), and normal or loss of *MCL1*. Table 1 shows the relationships between *MCL1* status and the clinicopathological parameters in ESCC. Sex, age, grade, invasive depth, vessel involvement, nerve involvement, lymph node metastasis, tumor site, smoking and clinical stage were not statistically correlated with high MCL1 gain (*P*>0.05).

### Survival outcomes

The median follow-up period was 33.0 months (95 % CI 38.33–45.25). There was 154 (58.8%) disease progression documented, and 149 patients (56.9%) died of ESCC during the follow up.

To clarify whether the *MCL1* gain could have a prognostic value, univariate and multivariate survival analyses were performed in all cases. Our univariate analysis revealed that *MCL1* gain wasn't associated with postoperative outcome (Figure 1). The invasive depth, vessel involvement, lymph node metastasis and clinical stage were significantly associated with postoperative outcome. In the multivariate analysis, lymph node metastasis (HR: 3.236, *P*<0.001 for DFS; HR: 3.501, *P*<0.001 for OS) and clinical stage (HR: 3.388, *P*<0.001 for DFS; HR: 3.616, *P*<0.001 for OS) were identified as independent worse prognostic factors as shown in Table 2.

**Table 2:** Univariate and mutivariate survival analysis for disease-free survival and overall survival in full cohort of ESCC

	DFS		OS	
	*P* value	Hazard ratio (CI 95%)	*P* value	Hazard ratio (CI 95%)
**Univariate analysis**				
Sex	0.825	1.049 (0.684-1.611)	0.787	1.062 (0.686-1.644)
Age	0.412	1.145 (0.829-1.583)	0.366	1.163 (0.838-1.615)
Grade	0.137	1.272 (0.927-1.747)	0.204	1.232 (0.893-1.701)
Invasive Depth	0.003	1.607 (1.174-2.199)	0.001	1.738 (1.251-2.415)
Vessel involement	0.001	1.830 (1.266-2.643)	0.001	1.937 (1.333-2.814)
Nerve involvement	0.948	0.989 (0.697-1.403)	0.877	0.972 (0.678-1.393)
Lymph node metastasis	<0.001	3.236 (2.307-4.540)	<0.001	3.501 (2.477-4.947)
Site	0.097	0.803 (0.620-1.041)	0.157	0.825 (0.633-1.076)
Clinical stage	<0.001	3.388 (2.447-4.691)	<0.001	3.616 (2.597-5.036)
Smoking	0.320	1.175 (0.855-1.615)	0.236	1.216 (0.880-1.679)
High *MCL1* gain	0.948	0.989 (0.697-1.403)	0.877	0.972 (0.678-1.393)
**Mutivariate analysis**				
Invasive Depth	0.350	1.181 (0.833-1.672)	0.219	1.258 (0.872-1.815)
Vessel involement	0.999	1.000 (0.676-1.479)	0.949	1.013 (0.681-1.508)
Lymph node metastasis	0.005	2.001 (1.236-3.240)	0.002	2.169 (1.331-3.533)
Clinical stage	0.010	1.929 (1.173-3.172)	0.013	1.891 (1.142-3.132)

### Survival analyses based on lymph node status

**Figure 2:**
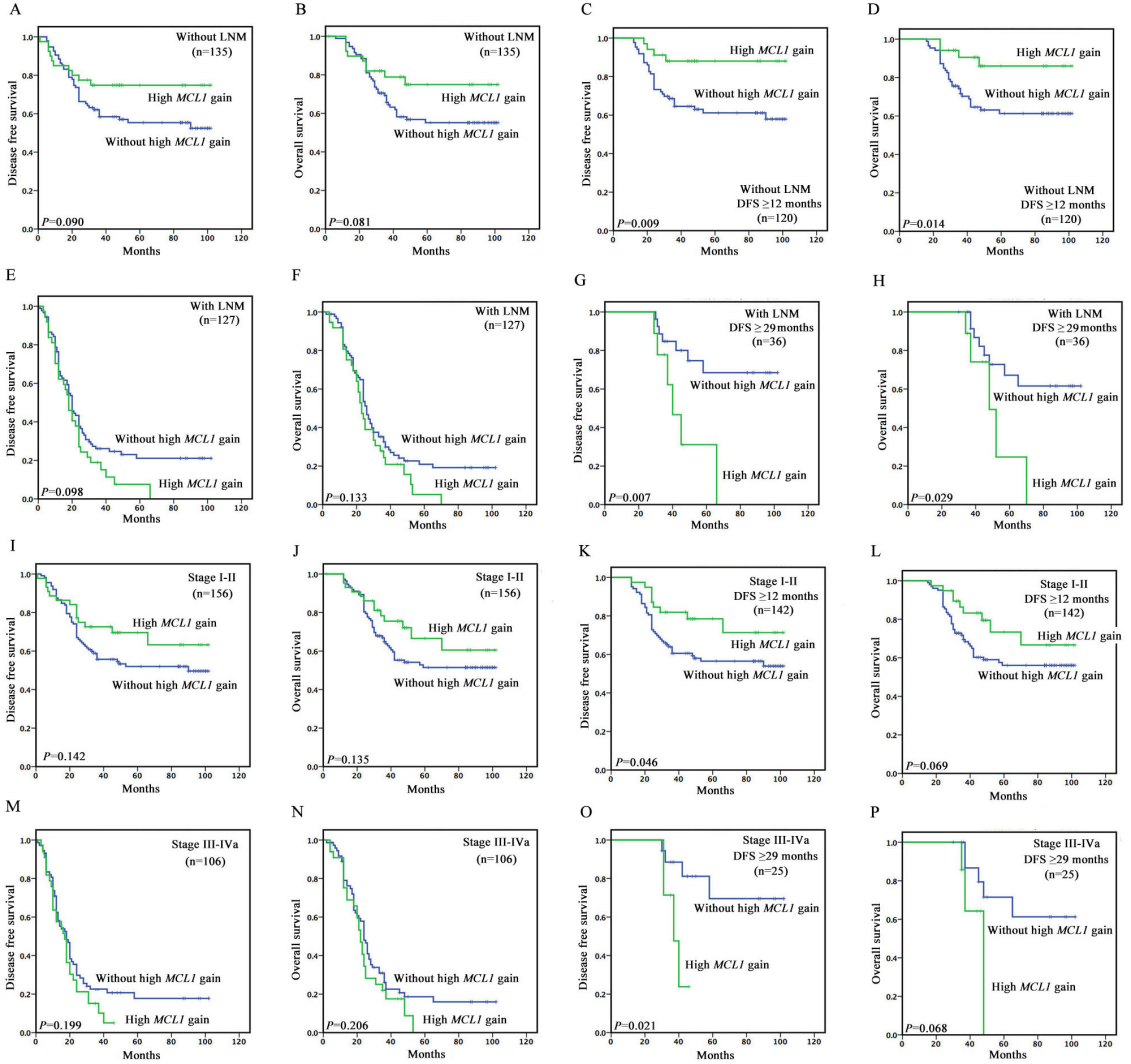
Kaplan–Meier survival curves illustrating prognostic effects of high *MCL1* gain in different subgroup of ESCC patients (**A** and **B**) In patients without lymph node metastasis (n=135), high *MCL1* gain tended to associate with better DFS (*P*=0.090) and OS (*P*=0.081). (**C** and **D**) In patients without lymph node metastasis and with disease free survival time greater than or equal to 12 months (n=120), high *MCL1* gain was associated with better DFS (*P*=0.009) and OS (*P*=0.014). (**E** and **F**) In patients with lymph node metastasis (n=127), high *MCL1* gain tended to associate with poorer DFS (*P*=0.098) and OS (*P*=0.133). (**G** and **H**) In patients with lymph node metastasis and with disease free survival time greater than or equal to 29 months (n=36), high *MCL1* gain tended to associate with poorer DFS (*P*=0.007) and OS (*P*=0.029). (**I** and **J**) In stage I-II patients (n=156), high *MCL1* gain tended to associate with better DFS (*P*=0.142) and OS (*P*=0.135). (**K** and **L**) In stage I-II patients with disease free survival time greater than or equal to 12 months (n=142), high *MCL1* gain tended to be associated with better DFS (*P*=0.046) and OS (*P*=0.069). (**M** and **N**) In stage III-IVa (n=106) patients, high *MCL1* gain tended to be associated with poorer DFS (*P*=0.199) and OS (*P*=0.206). (**O** and **P**) In stage III-IVa patients with disease free survival time greater than or equal to 29 months (n=25), high *MCL1* gain tended to associate with poorer DFS (*P*=0.021) and OS (*P*=0.068).

In patients with lymph node metastasis (n=127), high *MCL1* gain tended to associate with poorer DFS (*P*=0.098) and OS (*P*=0.133) (Figure 2). Among 37 patients with high *MCL1* gain, a poorer prognosis was observed, with a median DFS and OS of 18.0 and 23.0 months compared to 20.0 and 26.0 months for 90 patients without high *MCL1* gain. However, in patients without lymph node metastasis (n=135), high *MCL1* gain tended to associate with better DFS (*P*=0.090) and OS (*P*=0.081) (Figure 2).

**Figure 3:**
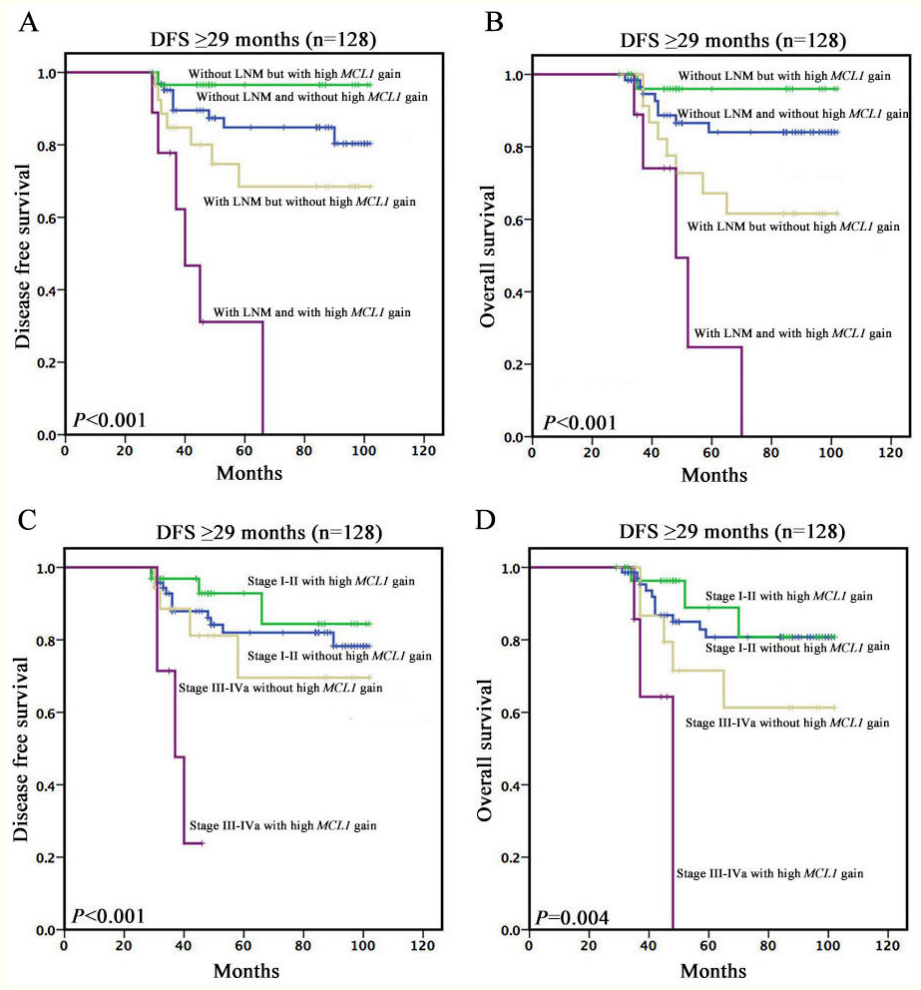
Kaplan–Meier survival curves among patients with disease free survival time greater than or equal to 29 months (**A** and **B**) Survivalanalyses based on lymph node status and high *MCL1* gain. (**C** and **D**) Survivalanalyses based on clinical stage and high *MCL1* gain.

Based on the primary survival curves, we made further analysis, and found significant time point for the bidirectional prognostic value of high *MCL1* gain. In patients without lymph node metastasis and with disease free survival time greater than or equal to 12 months (n=120), high *MCL1* gain was associated with better DFS (*P*=0.009) and OS (*P*=0.014) (Figure 2). In patients with lymph node metastasis and with disease free survival time greater than or equal to 29 months (n=36), high *MCL1* gain tended to associate with poorer DFS (*P*=0.007) and OS (*P*=0.029) (Figure 2 and 3) (Table 3). Among 9 patients with high *MCL1* gain, a significantly poorer prognosis was observed, with a median DFS and OS of 40.0 and 48.0 months compared to non-reached median survival for 27 patients without high *MCL1* gain.

**Table 3:** Univariate survival analysis for the prognostic significance of FGF4 amplification in different subgroup of ESCC patients

	DFS		OS	
	*P* value	Hazard ratio (CI 95%)	*P* value	Hazard ratio (CI 95%)
**Univariate analysis**				
Patients without LNM	0.099	0.559 (0.280-1.115)	0.090	0.534 (0.259-1.102)
Patients with LNM	0.111	1.400 (0.925-2.118)	0.144	1.370 (0.898-2.091)
Patients without LNM (<12 months)	0.399	1.615 (0.531-4.915)	0.250	2.049 (0.603-6.967)
Patients without LNM (≥12 months)	**0.016**	0.279 (0.099-0.788)	**0.022**	0.297 (0.105-0.842)
Patients with LNM (<12 months)	0.656	1.184 (0.563-2.488)	0.682	1.174 (0.545-2.532)
Patients with LNM (≥12 months)	0.146	1.452 (0.878-2.400)	0.173	1.426 (0.856-2.375)
Patients without LNM (<29 months)	0.553	1.252 (0.596-2.629)	0.552	1.272 (0.576-2.810)
Patients without LNM (≥29 months)	0.199	0.258 (0.033-2.043)	0.220	0.272 (0.034-2.181)
Patients with LNM (<29 months)	0.515	1.163 (0.738-1.832)	0.831	1.052 (0.662-1.672)
Patients with LNM (≥29 months)	**0.013**	4.206 (1.362-12.987)	**0.041**	3.288 (1.048-10.318)
I-II stage	0.151	0.649 (0.360-1.170)	0.143	0.634 (0.345-1.166)
III-IVa Stage	0.218	1.323 (0.848-2.065)	0.222	1.328 (0.842-2.096)
I-II stage (<12 months)	0.276	2.003 (0.575-6.978)	0.477	1.568 (0.453-5.421)
I-II Stage (≥12 months)	**0.054**	0.494 (0.241-1.013)	**0.077**	0.522 (0.254-1.073)
III-IVa stage (<12 months)	0.625	1.197 (0.581-2.467)	0.556	1.255 (0.589-2.672)
III-IVa Stage (≥12 months)	0.315	1.341 (0.757-2.375)	0.340	1.328 (0.742-2.378)
I-II stage (<29 months)	0.987	0.994 (0.506-1.952)	0.786	0.907 (0.451-1.827)
I-II Stage (≥29 months)	0.470	0.627 (0.176-2.228)	0.535	0.667 (0.185-2.398)
III-IVa stage (<29 months)	0.430	1.211 (0.753-1.948)	0.368	1.252 (0.767-2.044)
III-IVa Stage (≥29 months)	**0.037**	5.234 (1.105-24.798)	**0.099**	3.665 (0.785-17.115)

### Survival analyses based on clinical stage

In stage III-IVa (n=106) patients, high *MCL1* gain tended to be associated with poorer DFS (*P*=0.199) and OS (*P*=0.206) (Figure 2). Among 33 patients with high *MCL1* gain, a poorer prognosis was observed, with a median DFS and OS of 17.0 and 22.0 months compared to 18.0 and 24.0 months for 73 patients without high *MCL1* gain. However, in stage I-II patients (n=156), high *MCL1* gain tended to associate with better DFS (*P*=0.142) and OS (*P*=0.135) (Figure 2).

With the same analytic method we used in lymph node status mentioned above, we found similar results and time point as the lymph node status. In stage I-II patients with disease free survival time greater than or equal to 12 months (n=142), high *MCL1* gain tended to be associated with better DFS (*P*=0.046) and OS (*P*=0.069) (Figure 2). In stage III-IVa patients with disease free survival time greater than or equal to 29 months (n=25), high *MCL1* gain tended to associate with poorer DFS (*P*=0.021) and OS (*P*=0.068) (Figure 2 and 3) (Table 3). Among 7 patients with high *MCL1* gain, a significantly poorer prognosis was observed, with a median DFS and OS of 37.0 and 48.0 months compared to non-reached median survival for 18 patients without high *MCL1* gain.

## DISCUSSION

In the present retrospective study with FISH method, we investigated the clinicopathologic significance of *MCL1* copy number gain in ESCC. Herein, we firstly observed high *MCL1* copy number gain was bidirectional correlated with DFS and OS in ESCC patients with different lymph node status and clinical stage.

### The importance of *MCL1*

*MCL1* was discovered by Ruth Craig and colleagues in 1993, which was originally identified as a gene up-regulated early in the differentiation of a human myeloid leukemia cell line [14]. As the anti-apoptotic Bcl-2 family member, Mcl1 prevents pro-apoptotic proteins Bcl-2 homologous antagonist killer (Bak) and Bcl-2-associated protein X (Bax) from forming pores in the mitochondrial membrane. Then cytochrome c couldn't be released into the cytoplasm, which inhibits the subsequent activation of a family of cysteine proteases (caspases). Caspases are responsible for much of the macromolecular degradation observed during apoptosis [15]. Mcl1 has wide but particular tissue distribution, shown to be associated with the survival and development of diverse cell-types [16, 17]. Along with its roles in apoptosis and differentiation, Mcl1 is also known to influence cell cycle progression [18, 19]. An extensive genomic analysis of somatic copy number amplification (SCNA) in more than 3,000 cancer specimens representing 26 histological type, identified *MCL1* is enriched among regions of focal SCNA, and *MCL1* amplification is found in more than 10% of cancers across multiple tissue types, including breast cancer, lung adenocarcinoma and melanoma [13]. As previous study, Mcl1 contributes to tumorigenesis, particularly in solid cancers [20], and second generation Mcl1 antagonists are actively being sought [10, 21], distinguishing it as a potentially important molecular marker of tumor progress. However, to date and to the best of our knowledge, there have been rare studies addressing whether *MCL1* amplification develops in ESCC. In this study, we detected *MCL1* copy number variation in TMA from ESCC tumor tissues in a Chinese population, additionally, conducted survival analyses to analyze prognostic values of *MCL1* copy number gain on survival.

### *MCL1* amplification and FISH

Gene amplification can be detected by several methods, such as FISH, Southern blotting, Chromogenic *in situ* hybridization, Comparative genomic hybridization, and Real-time q-PCR. Most studies assessing *MCL* gene amplification have been performed using PCR techniques [8, 11]. It may suffer from normal cell contamination of the tumor sample, resulting in large fluctuations in copy number. FISH, removing the variable of normal cell contamination, has been generally accepted as the standard method for detection of gene amplification [22–24].

Therefore, we analyzed *MCL1* chromosomal alterations via the “gold standard”, FISH, in a cohort of ESCC patients. We found 29.4% of cases showed high copy number gain and 36.3% showed low copy number gain. The copy number analysis of 1q21.2 or *MCL1* locus inevitably raises important issues about how to define the ‘*MCL1* gain/amplification’ and whether to include the ‘low-level gain’ in the *MCL1* gain or not [8]. However, there is also no clear consensus as to the definition of *MCL1* amplification exanimated by FISH.

Given that *MCL1* gain criteria by using RT-PCR might not be directly applied to the FISH method in recent studies, *MYC* gain/ amplification criteria of FISH method was applied in our study. We found a poorer prognosis was observed in patients with high *MCL1* copy number gain, not low copy number gain. High *MCL1* copy number gain could lead to the aggressive biology of ESCC, it might be possible that the high gain of 1q21.2 or *MCL1* locus could enhance *MCL1* activity at certain level, which might be sufficient to effectively trigger amplification of transcription involving a various set of genes in tumor cells. However, the clinical meaning of *MCL1* high copy number gain or low copy number gain needs to be validated in prospective and larger scale study.

### The prognostic significance of *MCL1*

Lymph node metastasis is one of the major prognostic factors for esophageal cancer [25–28]. Some researchers, aiming to optimize the lymphadenectomy during esophagectomy for better survival, found that different patients with similar lymph node status may not share equal prognosis. They speculated some factors, together with lymph node metastasis, might contribute to the development and the progression of cancer [29–31].

Our study categorized the lymph node status with the combined analysis of *MCL1* copy number variation, and to examine this classification method in predicting the prognosis of ESCC patients. High *MCL1* copy number gain was found in 29.1% of 127 ESCC patients with lymph node metastasis and 29.6% of 135 ESCC patients without lymph node metastasis. High *MCL1* gain was associated with better survival in patients without lymph node metastasis 12 months later, and poorer survival in patients with lymph node metastasis 29 months later. In this study, we report for the first time that high *MCL1* gain was delayed bidirectional prognostic factor, and its prognostic significance was different in patients with different status of lymph node metastasis.

The prognostic significance of high *MCL1* gain was also compared in both stage I-II and III-IVa patients. We further certified that high *MCL1* gain was delayed prognostic factor, and similar to lymph node status, its prognostic significance was different in earlier stage and later stage ESCC patients. The mechanism research and external validations need to be extensively investigated in the future.

In conclusion, we observed that high *MCL1* gain was poorer prognostic factor for DFS and OS in later stage of ESCC patients (with lymph node metastasis or stage III-IVa) 29 months later. However, it was better prognostic factor for DFS and OS in earlier stage of ESCC patients (without lymph node metastasis or stage I-II) 12 months later. These findings might provide *MCL1* as the useful way of detailed risk stratification in patients with ESCC, and an insight into pathogenesis and mechanism of progression in ESCC.

## MATERIALS AND METHODS

### Patients and samples

A total of 262 ESCC patients were enrolled in this retrospective study. All patients had undergone primary surgical resection (radical transthoracic or transhiatal esophagectomy with lymphadenectomy) at Zhongshan Hospital, Fudan University between 2007 and 2010. None of these patients had received prior anti-tumor therapy (neither chemotherapy nor radiochemotherapy). The study was reviewed and approved by the local institution's Ethics Committee in accordance with the Declaration of Helsinki. Written informed consent was obtained from each patient for surgical specimen analyses.

The clinicopathological characteristics such as age, sex, smoking, location, and clinical stage were obtained from medical records and pathology reports. Hematoxylin and eosin (HE)-stained slides were reviewed by two pathologists to determine the histological subtypes, differentiation, invasion depth, lymph node metastasis, vessels and nerve involvement.

### Tissue microarrays (TMA)

The TMA blocks were manufactured as previously described [32]. Briefly, HE-stained slides were reviewed and the representative areas of interest with a high density of tumor cells were circled. The corresponding regions were marked on archival formalin-fixed, paraffin-embedded (FFPE) tissue blocks. The core of 2 mm wide and 6 mm long was extracted, vertically planted into the recipient block and then aggregated on the aggregation instrument.

### Fluorescence *in situ* hybridization (FISH)

To evaluate the copy number of *MCL1*, FISH assay was performed on the TMA sections of 5 mm thickness by using *MCL1* probe (Abbott Molecular, Abbott Park, IL, USA) that hybridize to 1q21.2 (*MCL1*) with spectrum gold signal, and Hybridization instrument (Abbott Molecular), according to manufacturer's instruction as previously described [33].

The FISH slides were interpreted by two independent and certified pathologists without information about the clinicopathologic characteristics. Tumor tissue was scanned to detect hot spots for *MCL1* copy numbers by using ×400 magnification. If the *MCL1* signals were homogeneously distributed, then random areas were selected to count the signals. Twenty non-overlapping tumor nuclei from three hot spots or random areas (60 nuclei per case) were evaluated, and the numbers of *MCL1* signals were counted at ×1000 magnification. An identical protocol is used at our institution for the evaluation of *MYC* copy number variation [34], with respect to the number of counted nuclei: (1) high *MCL1* gain (>5.0 average *MCL1* gene copies/nucleus); (2) low *MCL1* gain (2.5 to 5 average *MCL1* copies/nucleus); and (3) normal or loss of *MCL1* (<2.5 average *MCL1* copies/nucleus).

### Statistical analysis

Overall survival (OS) was defined as the time from surgery to the date of death from esophageal cancer; patients who were not reported as having died at the time of the analysis were censored at the date they were last known to be alive. Disease free survival (DFS) was defined as the time from surgery to first local, regional, or distant recurrence or death from any cause, whichever came first. Patients who were alive and did not experience recurrence at the time of the analysis were censored at the last disease assessment date.

The association between the clinicopathologic features and *MCL1* status was analyzed using the chi-square or Fisher’ s exact test, as appropriate. The patients’ survival was analyzed by using the Kaplan-Meier method and the log-rank test was used to determine if there were any significant differences between the survival curves. Univariate and multivariate regression analyses were performed by using Cox's proportional hazards model to determine the hazard ratio and 95% confidence intervals for each factor. *P* values<0.05 was considered as statistically significant (two-tailed). All statistical analyses were performed by SPSS21.0 (SPSS Inc, Chicago, IL, USA).
